# Modeling the prevalence of immunodeficiency-associated long-term vaccine-derived poliovirus excretors and the potential benefits of antiviral drugs

**DOI:** 10.1186/s12879-015-1115-5

**Published:** 2015-09-17

**Authors:** Radboud J. Duintjer Tebbens, Mark A. Pallansch, Kimberly M. Thompson

**Affiliations:** Kid Risk, Inc., 10524 Moss Park Rd., Ste. 204-364, Orlando, FL 32832 USA; Division of Viral Diseases, National Center for Immunization and Respiratory Diseases, Centers for Disease Control and Prevention, Atlanta, GA USA

## Abstract

**Background:**

A small number of individuals with B-cell-related primary immunodeficiency diseases (PIDs) may exhibit long-term (prolonged or chronic) excretion of immunodeficiency-associated vaccine-derived polioviruses (iVDPVs) following infection with oral poliovirus vaccine (OPV). These individuals pose a risk of live poliovirus reintroduction into the population after global wild poliovirus eradication and subsequent OPV cessation. Treatment with polio antiviral drugs may potentially stop excretion in some of these individuals and thus may reduce the future population risk.

**Methods:**

We developed a discrete event simulation model to characterize the global prevalence of long-term iVDPV excretors based on the best available evidence. We explored the impact of different assumptions about the effectiveness of polio antiviral drugs and the fraction of long-term excretors identified and treated.

**Results:**

Due to the rarity of long-term iVDPV excretion and limited data on the survival of PID patients in developing countries, uncertainty remains about the current and future prevalence of long-term iVDPV excretors. While the model suggests only approximately 30 current excretors globally and a rapid decrease after OPV cessation, most of these excrete asymptomatically and remain undetected. The possibility that one or more PID patients may continue to excrete iVDPVs for several years after OPV cessation represents a risk for reintroduction of live polioviruses after OPV cessation, particularly for middle-income countries. With the effectiveness of a single polio antiviral drug possibly as low as 40 % and no system in place to identify and treat asymptomatic excretors, the impact of passive use of a single polio antiviral drug to treat identified excretors appears limited. Higher drug effectiveness and active efforts to identify long-term excretors will dramatically increase the benefits of polio antiviral drugs.

**Conclusions:**

Efforts to develop a second polio antiviral compound to increase polio antiviral effectiveness and/or to maximize the identification and treatment of affected individuals represent important risk management opportunities for the polio endgame. Better data on the survival of PID patients in developing countries and more longitudinal data on their exposure to and recovery from OPV infections would improve our understanding of the risks associated with iVDPV excretors and the benefits of further investments in polio antiviral drugs.

## Introduction

The Global Polio Eradication Initiative (GPEI) plans to stop the use of the oral poliovirus vaccine (OPV) after assurance of global interruption of wild poliovirus (WPV) transmission to eliminate the risks of poliomyelitis disease associated with continued OPV use [1, 2]. Given the apparent global interruption of indigenous serotype 2 WPV (WPV2) by 2000 [3], the GPEI Strategic Plan 2013-2018 [4] calls for phased, but coordinated cessation of the three OPV serotypes, starting with coordinated cessation of serotype 2-containing OPV (OPV2 cessation) in April 2016 [5]. The risks associated with OPV use include the relatively predictable cases of vaccine-associated paralytic poliomyelitis (VAPP) in a small fraction of OPV recipients and close contacts, the emergence of circulating vaccine-derived polioviruses (cVDPVs) in populations with low immunity to poliovirus transmission that can behave like WPVs and cause outbreaks, and possible reintroduction of immunodeficiency-associated vaccine-derived polioviruses (iVDPVs) from rare individuals with B-cell-related primary immunodeficiencie diseases (PIDs) who can continue to excrete polioviruses for years [2]. In addition, after cessation of one or more OPV serotypes, any OPV of the withdrawn serotype(s) that mistakenly remains in the field or insufficiently contained in laboratories and vaccine production sites poses a risk of reintroducing a live poliovirus (LPV) in increasingly susceptible populations [6]. For any given serotype, new recipient VAPP cases of that serotype in immunocompetent individuals will almost immediately stop after OPV cessation, but cVDPVs can still evolve from continued circulation of OPV-related strains and get recognized through paralytic cases within about a year or so of OPV cessation in populations with insufficient immunity at the time of OPV cessation [7]. If the world can successfully control these cVDPV outbreaks, then long-term iVDPV excretors represent the primary OPV-associated risk of outbreaks.

While any cVDPVs would likely emerge at a time of relatively high population immunity to transmission given the recent use of OPV in most countries [7], after OPV cessation, countries will only use the inactivated poliovirus vaccine (IPV) for polio immunization. While IPV appears to provide sufficient population immunity to prevent transmission in populations with high hygiene levels and low fecal-oral transmission [8–10], recent asymptomatic circulation of serotype 1 WPV (WPV1) in parts of Israel despite very high IPV routine immunization coverage strongly suggests that IPV-induced immunity alone cannot prevent or stop transmission in some settings [11, 12]. Modeling further suggests that population immunity to poliovirus transmission will drop significantly and fairly quickly after OPV cessation in most developing countries, regardless of IPV use due to both new birth cohorts that only receive IPV and waning immunity in those previously immunized or exposed to LPVs [13]. Strategies to respond to and control any iVDPV-associated outbreaks in developing countries long after OPV cessation require careful consideration, because using OPV could re-introduce large amounts of LPV at a time of low population immunity, and IPV may not provide sufficient population immunity to stop its transmission. Recognizing this risk, in 2006 an Institute of Medicine committee recommended the development of one or more polio antiviral drugs (PAVDs) [14]. Currently, one compound (pocapavir) exists with proven ability to stop poliovirus excretion in a fraction of immunocompetent individuals based on one small clinical trial, and other compounds remain in development [15].

We previously estimated the prevalence of long-term iVDPV excretors (i.e., defined as either prolonged excretors with between 6 months and 5 years of excretion, or chronic excretors with over 5 years of excretion) and associated probabilities of post-OPV-cessation outbreaks based on the information available as of late 2005 [2]. The analysis relied on limited studies that observed no long-term iVDPV excretors among 384 persons with PIDs studied [16, 17] and knowledge about 4 identified asymptomatic and active long-term excretors [2]. We accounted for uncertainty by characterizing a wide distribution of the risk that included an upper bound for the prevalence of long-term excretors of 140 in upper middle- and high-income countries before OPV cessation [2]. The probability distribution for the ratio of reported to actual long-term excretors reflected the findings from screening studies [16, 17] and an estimate of approximately 1:100,000 people affected with agammaglobulinemia, which represents only one of the many PIDs that can lead to long-term excretion. The 2006 analysis [2] emphasized the need for additional studies that would reduce uncertainty.

Since 2006, the GPEI intensified the search for asymptomatic long-term iVDPV excretors through screening studies and for paralytic long-term iVDPV excretors through the acute flaccid paralysis surveillance system. Consistent with this intensification, the average annual number of identified long-term excretors increased by a factor of 2.5 from 2000–2005 to 2006–2013 [18]. Improvements in the quality of health care delivery in middle - income countries may also imply increased survival of PID patients and affect the prevalence of long-term excretors. The increased number of individuals with PIDs screened for long-term iVDPV excretion led to some identified long-term excretors, and the updated data provide better information to characterize the bounds of the proportion of PID patients with long-term iVDPV excretion [19–21].

The accumulation of new evidence about PID prevalence, survival, and long-term excretion of polioviruses and the parallel development of PAVDs motivate a re-analysis of the iVDPV risks going forward and consideration of the potential impact of PAVDs. In addition, the serotype-specific OPV vaccination and cessation strategies that did not exist at the time of our prior analysis motivate the consideration of serotype-specific differences in iVDPV risks [22]. We developed a stochastic, discrete-event simulation (DES) model to estimate the prevalence of long-term iVDPV excretors up until and after OPV cessation based on current evidence. The model incorporates the available new evidence about iVDPVs and explores the potential impact of PAVDs on iVDPV prevalence for use in integrated global modeling of long-term poliovirus risk management policies [23].

## Background

Immunocompetent individuals infected with poliovirus typically excrete for several weeks (mean around 30 days) and no longer than 3 months [8, 9, 24, 25]. However, a small fraction of individuals with B-cell-related PIDs can excrete for longer [26–30]. The GPEI maintains a registry of known long-term excretors that included 73 immunodeficient patients with evidence of more than 6 months of poliovirus excretion identified prior to 2014 [18], which largely overlaps with the 68 long-term poliovirus excretors listed in a recent review [31]. Of the 73 known long-term excretors, 13 (17 %) presented with common variable immunodeficiency disease (CVID), while the remainder presented with other PIDs (oPIDs), including not further specified PIDs (16), severe combined immunodeficiency disease (SCID)(14), hypogammaglobulinemia (11), X-linked agammaglobulinemia (8), agammaglobulinemia (6), major histocompatibility complex class II molecule deficiency (2), humoral and cellular immunodeficiency (1), HLA-DR-associated immunodeficiency (1), and immunodeficiency-centromeric instability-facial abnormalities (1). Six of the 13 (36 %) long-term excretors with CVID fit the criteria for chronic excretion [29, 30, 32–34] and one additional CVID patient died approximately 5 years after the probable onset of infection [35], while none of the long-term excretors with oPIDs excreted longer than 5 years.

The overall prevalence of PIDs globally remains highly uncertain due to the large number of PID conditions, differences in case definitions, and variability between countries in both genetic profiles (e.g., consanguinity) and survival rates of PID patients [16, 36, 37]. Estimates of the prevalence of CVIDs reported to PID registries generally range from 1:10,000 to 1:100,000 people with a best estimate around 1:50,000 people for high-income countries [18]. One US telephone survey of self-reported PIDs without clinical verification reported 8 CVID and 15 oPID patients from approximately 10,000 surveyed households representing approximately 27,000 people. These numbers translate into prevalence rates of CVIDs and oPIDs of approximately 1:3,300 and 1:1,800 people, respectively [38]. However, the study concludes that “the true incidence and prevalence of these conditions will never be known until there is newborn or population screening for these defects [38], p. 501.” Notably, other authors extrapolated these results globally without any adjustments for study bias or differential survival of PID patients in different countries and estimated an unrealistic global prevalence as up to 6 million PID patients worldwide [39]. This estimate contrasts sharply with the approximately 77,000 PID patients reported in a survey among physicians in 225 Jeffrey Modell Foundation Centers in 78 countries, which included approximately 8600 CVID patients [40]. With respect to oPIDs, one Minnesota county with a comprehensive medical record system estimated an overall PID incidence for 2001–6 of 10.3 per 100,000 person-years, with an age-specific incidence of approximately 22 per 100,000 person-years aged 0–5 years [41]. These rates do not directly translate into rates per newborn due to imperfect diagnosis and time delays associated with development of symptoms and diagnosis. A study of VAPP in the US assumed an incidence of approximately 1 PID patient per 10,000 births in the US [42].

Intravenous immunoglobulin (IVIG) therapy reduces morbidity and probably also mortality. Patients with CVIDs can survive for many years with appropriate and uninterrupted IVIG therapy, although they experience higher death rates than the general population even in developed countries [36, 37, 41, 43, 44]. The life expectancy of other types of PIDs varies [36], with more severe forms (e.g., SCID) rarely surviving beyond 1 year of age in developing countries [20], and less severe forms with life expectancy more similar to CVID patients (e.g., patients with other hypogammaglobulinemia). The onset of symptoms varies widely among CVID patients, with an average of approximately 25 years [43–45], while severe oPIDs most commonly associated with long-term poliovirus excretion typically occur earlier in life. Over the past 25 years, IVIG therapy has become the standard of care for PID patients in developed countries [46], and some developing countries recently began providing IVIG to identified PID patients through their health care systems [20]. The global supply of IVIG remains limited [46] and effective treatment requires consistent and high quality IVIG administration, with any disruptions exposing patients to a risk of developing infectious disease complications [34].

Although the acute flaccid paralysis surveillance system cannot detect individual asymptomatic long-term excretors, the GPEI registry identified 16 asymptomatic long-term excretors through other sources (primarily known PID patients in higher-income countries identified by the treating physician). In addition, environmental surveillance activities in several countries identified 9 highly divergent VDPVs likely from different immunodeficient chronic excretors in sewage, but they could not link the detected viruses to any individuals with PIDs [18, 47–50]. The detection of these viruses suggests the existence of significantly more asymptomatic long-term iVDPV excretors than the 16 individuals known to the GPEI.

Table 1 summarizes the results of several screening studies that provided additional evidence about the frequency of long-term poliovirus excretion. All studies combined that screened PID patients for long-term excretion detected only 1 prolonged excretor (a CVID patient in Sri Lanka) out of 318 CVID patients (0.3 %) and 978 total PID patients (0.1 %) (Table 1). Another child in Sri Lanka with SCID died while infected and appeared as a long-term excretor in some publications assuming infection following the first OPV dose received [18, 35, 51] but not in other publications based on the duration of documented excretion [20, 52], which reflects uncertainty about the date of the OPV exposure that initiated the infection. Given the low numbers of observations overall, inclusion of this excretor would double the apparent rate of long-term poliovirus excretors per PID patient (e.g., from 1 in 978 to 2 in 978). A 7-country screening study identified 17 total poliovirus excretors and followed these individuals longitudinally until they stopped excreting or died. In this study, 6 excretors died within 6 months while still excreting poliovirus. Of the remaining 11 excretors, 10 stopped excreting spontaneously within 6 months, while 1 spontaneously stopped excreting after 8 months [52].

**Table 1:** Results of screening studies for long-term iVDPV excretion among individuals with PIDs

Country	Total patients with PIDs studied	CVID patients studied	Poliovirus excretors	iVDPV excretors	Patients with > 6 months of documented excretion	Source(s)
Bangladesh	13	0	1	0	0	[52]
Brazil	95	70	3	0	0	[16]
China	167	22	3	0	0	[52]
Egypt^a^	15	3	2	0	0	[21]
Iran	43	16	1	0	0	[52]
Italy	38	0	0	0	0	[17]
Mexico	33	5	1	0	0	[16]
Philippines	70	6	1	0	0	[52, 60]
Russia	136	27	0	0	0	[52]
Sri Lanka	51	13	5	2	1^b^	[20, 52]
Tunisia	16	2	4	0	0	[61]
Tunisia	82	14	6		0	[19, 52]
United Kingdom	125	65	0	0	0	[16]
United States	94	75	0	0	0	[16]
Total	978	318	27	2	1	

^a^Preliminary results

^b^The prolonged iVDPV excretor was diagnosed a CVID patient

PID patients with poliovirus infections remain vulnerable to VAPP, and the GPEI identified most long-term excretors when they presented with VAPP (i.e., 57 of 73, 78 %) [18]. A retrospective analysis of all 37 immunodeficient VAPP cases reported in the United States between 1975 and 1997 found 6 months of excretion or more for 6 of 31 (19 %) patients with follow-up samples available, including 1 chronic excretor with CVID [53]. Some patients developed fatal VAPP after a long period of excretion, while others survived and continued to excrete iVDPVs long after VAPP onset, and both asymptomatic long-term excretors and those with VAPP may spontaneously clear the infection [2, 18, 53]. For most known long-term excretors, we could not determine their IVIG therapy status at the time of OPV infection, but the majority of those with available data appeared to have acquired the OPV infection before the start of IVIG therapy. However, at least 2 excretors started IVIG therapy before the estimated onset of the long-term OPV infection [20, 34]. Thus, IVIG therapy may reduce the probability of acquiring an OPV infection or of an OPV infection establishing persistent replication, but it does not completely prevent long-term infection.

## Methods

### Stages of PID disease and OPV infection

**Fig. 1:**
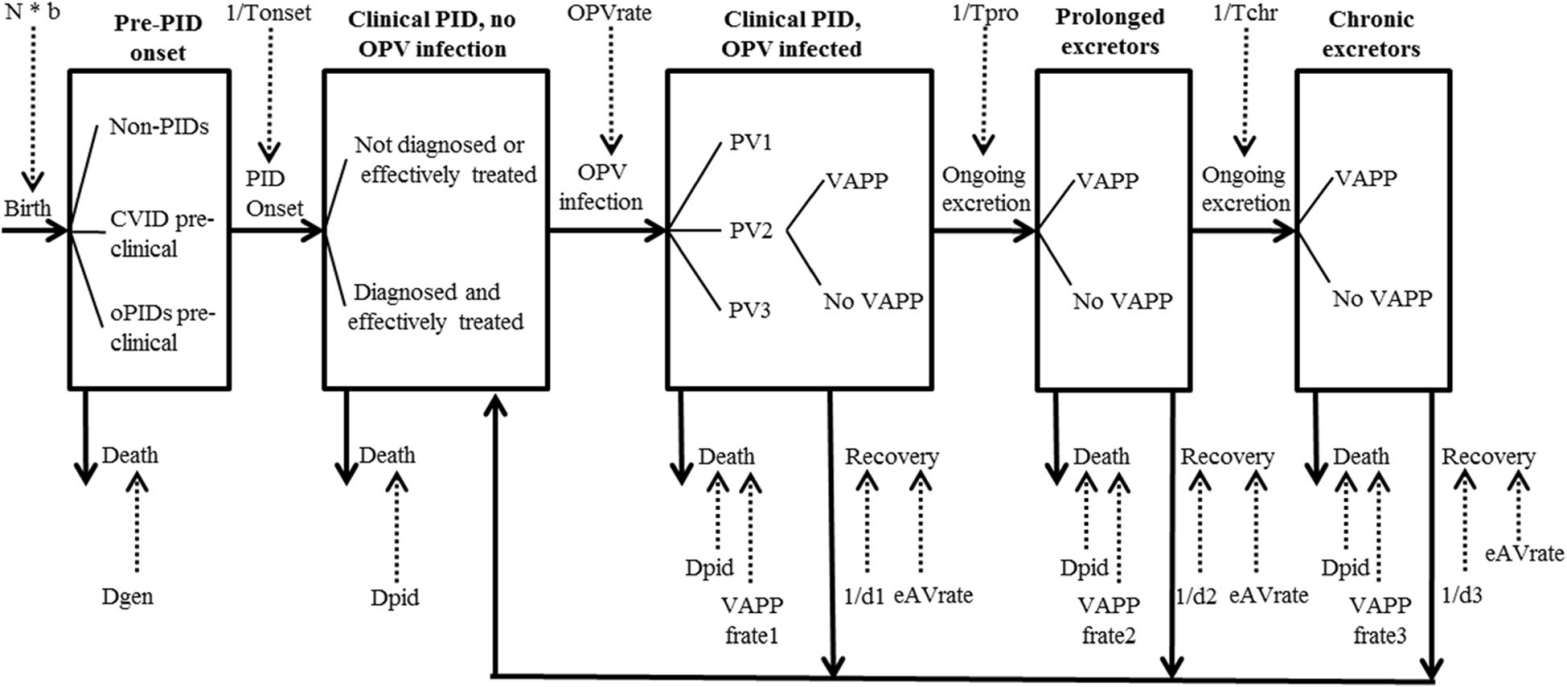
Conceptual diagram of states for individuals in the population with respect to the development of various stages and types of long-term iVDPV excretion. Arrows between boxes indicate flows that represent PID disease and poliovirus infection progression, while trees within boxes represent branching between distinct pathways that imply one or more different downstream rates and probabilities. Notation (see also list of abbreviations): b, birth rate; d1, duration of infection for clinical PID patients with typical OPV infection; d2, duration of infection for prolonged excretors; d3, duration of infection for chronic excretors; Dgen, death rate for general population (by age); Dpid, death rate for clinical PID patients (by, PID category and receipt of effective treatment); eAVrate, effective rate of PAVD use; N, population size; OPVrate, combined primary (i.e., vaccination) and secondary OPV infection rate (by age, OPV use over time, diagnose status, and IVIG rate); Tchr, time to move from prolonged to chronic infection; Tonset, average time to onset of clinical PID; Tpro, time to move from OPV to prolonged infection; VAPPfrate(1, 2, and 3), VAPP fatality rate for PID patients (during OPV infection, prolonged excretion, and chronic excretion, respectively)

Figure 1 provides our conceptual diagram of the progression of PID patients through various clinical and poliovirus excretion stages (large boxes) with inflows and outflows from the stages indicated by arrows with solid lines. The arrows with dotted lines indicate model inputs that influence the flows. The accumulation of individuals in the stages provides the overall prevalence in the population, with the total number in the two stages on the right representing the prevalence of long-term (i.e., prolonged and chronic) excretors. We include trees with branches within some of the stages to indicate stratification of stages into different groups that may experience different outflows going forward. For example, the death rate for clinical PIDs depends on the type of PID and effective treatment with IVIG. Death rates, treatment probabilities, and OPV infection rates further change over time and vary between countries. Infected patients may develop VAPP at any time as they move through the OPV, prolonged, and chronic infection stages, so long as the infection continues and the individuals survive. Figure 1 suggests that once individuals recover from infection, they can potentially become re-infected. No known cases of longterm re-infection after recovery from a long-term infection exist, but some evidence exists of many repeated poliovirus and other enterovirus infections in a PID patient, with one poliovirus infection of at least 4 months [54].

### Discrete-event simulation model

The population of long-term excretors changes over time, and consequently modeling the risks should involve appropriate characterization of the dynamics of accumulation of excretors and their progression through different stages of infection until they ultimately either stop excreting spontaneously or die. Based on our understanding of how PID patients move through various clinical and OPV infection stages (Fig. 1), we developed a DES model to estimate long-term poliovirus excretor prevalence over time following each PID patient for life. The model uses a discrete time step of 1 month. We use the stratification from an integrated global model (i.e., the global model) that assigns all countries to epidemiological blocks of approximately 100 million people in 2013 classified by polio vaccine use as of 2013 (i.e., OPV-only, sequential IPV/OPV, or IPV-only), geographic proximity, and economic status [23, 55]. We estimated poliovirus basic reproduction number (R_0_) values for each block based on prior modeling experience and use R_0_ as a proxy for many factors that affect poliovirus transmission and the health system quality by correlating R_0_ with vaccine coverage and take rate, extent of fecal-oral transmission, and costs. Table 2 provides the resulting global R_0_ distribution for the different strata of income levels and polio vaccine use as of 2013. Because the accumulation of PID patients increases relatively slowly in the model, we use a long burn-in period, assuming the same general population death rates throughout, but increasing treatment fractions (see below). Table 2 includes the assumptions about when IPV-only or IPV/OPV starts during the burn-in period for different income levels.

**Table 2:** Assumed distribution of wild poliovirus serotype 1 R_0_ values for the different income level and current polio vaccine use strata, as used in the global model, and assumed simplified burn-in period [23]^a^

Income level (as of 2013)	Polio vaccine(s) used (as of 2013)	Population size in billions (as of 2013)	Years until OPV use starts^b^	Year until IPV-only or IPV/OPV starts^b^	Number of assigned blocks	Number of blocks assigned to each R_0_ (for WPV1)									
						4	5	6	7	8	9	10	11	12	13
Low	OPV-only	0.86	33	N/A	8	0	0	0	0	0	1	3	2	1	1
Lower middle	OPV-only	2.48	33	N/A	25	0	0	1	4	5	2	0	10	1	2
Upper middle	OPV-only	1.87	20	N/A	19	0	0	1	16	2	0	0	0	0	0
Upper middle	IPV/OPV	0.50	20	58	7	0		2	3	2	0	0	0	0	0
High	IPV/OPV	0.23	10	50	2	0	0	0	2	0	0	0	0	0	0
High	IPV-only	1.02	10	50	10^c^	2	7.7	0.3	0	0	0	0	0	0	0

^a^Does not include countries with unspecified income level totaling approximately 23 million people and two high-income countries that still use OPV-only totaling approximately 34 million people (all as of 2013); Assigns 67 million people in IPV/OPV-using lower middle-income countries to IPV/OPV-using upper middle-income blocks, 74 million people in IPV-only using upper middle-income countries to IPV-only-using high-income blocks, and 23 million people in OPV-only-using high-income countries to OPV-using lower or upper middle-income blocks

^b^Years relative to beginning of burn-in period 65 years ago (i.e., 1950)

^c^Includes some blocks with subpopulations assigned to different R_0_ values

### Births and characteristics determined at birth

Table 3 presents the events in the DES model, with estimates of the probabilities based on the limited available evidence and judgment. For each block, we generate the monthly number of births (b) over time based on demographic data [56]. Using the estimated fraction of births with PIDs (Ppid), the model generates the total number of newborns in each month with a genetic predisposition of each PID category (CVID or oPIDs) using a random draw from a Poisson distribution with rate b × Ppid. The model differentiates CVID from oPIDs because it assumes that only CVID patients may develop chronic poliovirus excretion. This level of stratification combines all oPIDs into one category, despite the wide spectrum of conditions, disease severity, treatments, survival, and possibly also different abilities to become infected and/or clear poliovirus, because insufficient data exist to separately model all PID defects. At birth, we also randomly determine whether each PID patient will become a long-term excretor if infected with OPV after onset of clinical symptoms resulting from the PID (upper section of Table 3). The probability of potential long-term excretion (i.e., if infected with OPV after PID onset and surviving long enough) represents a key uncertain input. Although we do not know whether individual host properties or random events associated with the OPV infection (e.g., the replication site) determine the ability to excrete long-term, we assume a pre-determined patient-specific ability to excrete long-term, if surviving long enough and infected after clinical PID onset. In the absence of resource constraints and practical considerations, the ideal screening study would follow a large number of PID patients over many years after PID onset to determine when they become infected with OPV and when they recover. The existing screening studies in Table 1 only provide a cross-section of PID patients and identified individuals excreting at a given point in time, with limited follow-up of only those found excreting. Depending on the interpretation of the data discussed above, estimates of the rate of long-term excretion per PID patient may range from approximately 0.1 % (i.e., the proportion of all PIDs found to excrete poliovirus during follow-up of cross-sectionally screened PID patients) [52] to approximately 10 % (i.e., the proportion of surviving patients with poliovirus excretion at time of cross-sectional screening found to excrete long-term) [52] or as high as almost 20 % (i.e., the proportion of surviving immunodeficient VAPP patients found to excrete long-term) [53]. The first estimate provides a lower bound of potential long-term excretors because it excludes excretors who did not yet become infected at the time of screening or who already stopped excreting. The 10 % estimate may represent an overestimate of the true proportion because its denominator skews towards long-term excretors (since they remain more likely to excrete poliovirus at the time of screening). The 20 % estimate provides an overestimate if VAPP is more common for long-term than typical excretors with a PID, which appears plausible given that long-term excretors cannot rapidly clear polioviruses and a longer infection implies a greater chance of the virus entering the central nervous system. We assume that 1 % of PID patients may become a prolonged excretor if infected and surviving (Table 3). Although close to half of the identified CVID patients with long-term excretion exhibit chronic excretion, we assume that relatively more prolonged than chronic excretors with CVID go undetected due to their shorter period of excretion, implying a smaller probability (0.5 %) of a CVID patient becoming a chronic excretor if infected and surviving.

**Table 3:** Inputs for the DES model of long-term poliovirus excretor prevalence

Model input	Base case value	Source	Notes
Births, by income level and polio vaccine use as of 2013 [1/month]	Varies with time	[23, 56]	Using number of 0-year old children as of 2013, divided by 12 months; stratification by income level and polio vaccine as of 2013 as in Table 2
Probabilities of attributes determined at birth			
PID pre-disposition		[41]	Based on reported annual total PID incidence in 0–5 year olds during 2000–2006, attributing 21 % to CVIDs based on total reported fraction of new PID cases, excluding IgA deficiency and transient hypogammaglobulinemia in infancy
- CVID	1/32,000		
- oPIDs	1/8,500		
Potential long-term excretion (if OPV-infected and surviving)		[18, 53] and Table 1	Assumes lower chance of becoming potential chronic than prolonged excretor for CVIDs based on limited observations of both; for oPIDs, no known cases of chronic excretion exist
- Prolonged, CVID or oPIDs	0.01		
- Chronic, CVID	0.005		
- Chronic, oPID	0		
Monthly event probabilities and related relative probabilities			
Death, general population, by income level	Varies with age	[56]	Based on 2013 estimates of annualized death rates; does not include fatal VAPP which may occur separately; applies prior to clinical PID onset
Death, PID patients	Varies with time since onset/treatment	[20, 37, 44]B^*^	Baseline monthly death rates calculated from survival curves (Fig. 2), then multiplied by relative monthly risk of death for R_0_ and treatment status; does not include fatal VAPP which may occur separately, and effect of treatment lapses, which subject otherwise treated PID patients to the untreated PID death rates for the duration of the lapse
Relative monthly death rate vs. baseline, by treatment status		B	Apply death rates as a function of time since treatment start for treated PID patients and since PID onset for untreated patients
- Treated	1		
- Untreated	5		
Relative monthly death rate vs. baseline, by R_0_		B	
- 4 or 5	1		
- 6	5		
- 7	10		
- 8	20		
- 9	25		
- 10	35		
- 11	40		
- 12	45		
- 13	50		
Treatment lapse		B	
- Low-income	0.8		
- Lower middle-income	0.75		
- Upper middle-income	0.1		
- High-income	0.001		
PID onset		[44, 45]	Corresponds to average of onset of approximately 25 (CVID) and 2 (oPIDs) years
- CVID	1/300		
- oPIDs	1/24		
Diagnosis		[43–45]	Corresponds to average diagnostic delay of approximately 5 (CVID) and 1 (oPID) years
- CVID	1/60		
- oPIDs	1/12		
Primary OPV infection, if OPV-only RI and not diagnosed with PID		B	Defined as monthly probability of infection with any serotype due to receipt of OPV; assumes 3 OPV infections during primary vaccination in first year of life, 1 infection from OPV booster dose during ages 1–4 in high-income countries, 1 annual infection from OPV supplemental immunization activity (SIA) doses during ages 1–4 in other countries, and no OPV doses after age 4
- Any income level, age 0	1/4		
- Not high-income, age 1-4	1/12		
- High-income,age 1-4	1/48		
- Any income level, age > 4	0		
Primary OPV infection, if IPV/OPV RI and not diagnosed with PID		B	Assumes 2 instead of 3 OPV infections during first year of life, with 1 additional infection during ages 1-4
- Any income level, age 0	1/6		
- Any income level, age 1-4	1/48		
- Any income level, age > 4	0		
Relative probability of primary OPV infection, diagnosed vs. not diagnosed	0.1	B	Assume contra-indications typically followed for vaccination
Relative probability of secondary OPV infection, diagnosed vs. not diagnosed	0.5	B	Assume siblings will sometimes avoid live vaccines
Relative probability of long-term OPV infection if treated vs. not treated	0.5	B	Assumes some effect of IVIG on the ability of an OPV infection to become persistent; excludes treated PID patients experiencing a treatment lapse
Secondary OPV infection, if OPV-only RI		[62, 63] B	Defined as probability of infection with any serotype due to secondary OPV exposure; Baseline rates based on approximately 45 % secondarily infected from OPV RI by age 20 months in US [62] and approximately 50 % secondarily infected from SIAs in Oman and Cuba [63, 64], assuming 1 SIA per year on average
- Not high-income, age 0-4	1/24		
- High-income, age 0-4	0.029		
- Not high-income, age 5-14	0.5 × 1/24		
- High-income, age 0-4	0.5 × 0.029		
- Not high-income, age > 14	0.25 × 1/24		
- High-income, age >14	0.25 × 0.029		
Relative probability of secondary OPV infection in any income level if IPV/OPV RI vs. high-income country with OPV-only RI	0.5	B	
Probability of serotype-specific OPV infection given any OPV infection before OPV2 cessation		[18]	Based on distribution of isolated serotypes from known long-term poliovirus excretors; see text
- Serotype 1	0.22		
- Serotype 2	0.62		
- Serotype 3	0.16		
Probability of serotype-specific OPV infection given any OPV infection, after OPV2 cessation		B	Assumes same relative distribution of serotypes 1 and 3 after OPV2 cessation as before OPV2 cessation; see text
- Serotype 1	0.58		
- Serotype 2	0		
- Serotype 3	0.42		
Recovery from OPV infection, by time since onset of infection		[2, 18, 65] B	Implies average duration of approximately 3 months for a “typical” infection (truncated at 6 months), which is somewhat longer than immunocompetent individuals, consistent with observations from Finland;[65] 2 years for a prolonged infection (truncated at 5 years), and 10 years for a chronic infection (not truncated)
- Typical, months 0-4	1/3		
- Typical, month 5	1		
- Prolonged, month 0-5	0		
- Prolonged, months 6-58	1/18		
- Prolonged, month 59	1		
- Chronic, month 0-59	0		
- Chronic, from month 60	1/120		
VAPP		[18, 42, 53] B	Based on US VAPP incidence by PID category and calibration to reported paralytic long-term excretors (see text); assumes no chance of VAPP if effectively treated (i.e., with IVIG), excluding during treatment lapse
- CVID, not treated	0.004		
- oPIDs, not treated	0.008		
- Any PID, treated	0		
Fatal VAPP		[53, 66]	Based on case-fatality rates among 6 immunodeficient VAPP patients in Iran and 36 immunodeficient VAPP cases in the US; for the latter, we assume that a death within a year of VAPP onset represents death associated with VAPP (even in the case of another cause of death indicated) due to comorbidity
- Low-income countries	0.5		
- Lower middle-income countries	0.4		
- Upper middle-income countries	0.3		
- High-income countries	0.14		

Notes: * B indicates estimate based on judgment

### PID survival and treatment

**Fig. 2:**
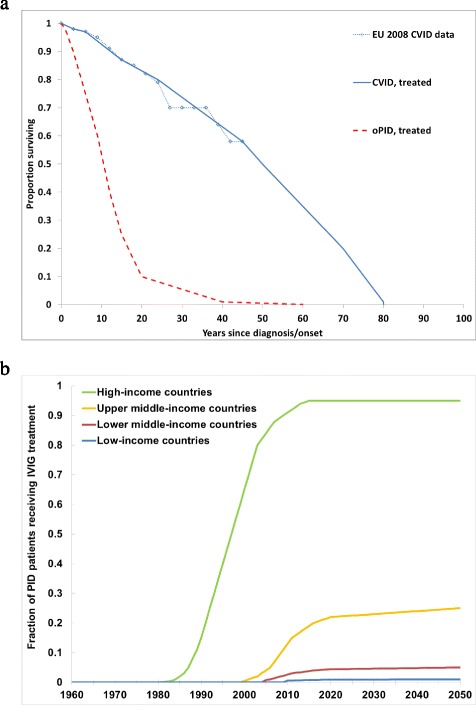
Assumed baseline survival curves for PID patients in populations with R_0_ values for WPV1 of 4 or 5 and assumed treatment fractions as a function of time, by income level. **a** Baseline survival curves, compared with reported survival for CVID patients in high-income countries [34]. **b** Fraction of PID patients treated with IVIG, based on literature [20, 26, 30, 31, 36, 45] and judgment

The remainder of Table 3 lists events and associated probabilities that may occur at monthly intervals over the lifetime of the PID patient, including death prior to clinical PID onset based on age-specific general population death rates in each income level [56]. After clinical PID onset, we assume different monthly probabilities of death depending on the PID category (i.e., CVID or oPID), treatment status, and R_0_ for WPV1 in the population in which the PID lives. Figure 2a shows the assumed baseline survival curves for effectively treated and not effectively treated PID patients, and Fig. 2b shows the assumed treatment fraction as a function of time in each income level. We constructed the baseline survival curve for treated CVID patient from a longitudinal study of CVID patients in Europe [44], which we assume applies for CVID patients living in populations with an R_0_ of 4 or 5. From the survival curve, we compute the monthly probability of death D(t1) between t1 and t2 months after CVID onset as D(t1) = 1-S(t2)/S(t1)^1/(t2-t1)^, where S(t1) and S(t2) represent the proportion surviving t1 and t2 months after CVID onset, respectively. For oPIDs, we constructed a baseline survival curve based on judgment and limited evidence that suggests very short survival for some oPIDs (e.g., SCID), but a relatively long tail due to some oPID defects with longer survival [36]. Based on data [44] that suggest much shorter survival prior to widespread IVIG treatment in high-income countries, we assume 5-fold higher monthly probabilities of death for CVID patients and an equal relative monthly risk of death for untreated compared to treated oPID patients (Table 3). We assume that even for treated PID patients, relative death rates increase with increasing R_0_, because higher poliovirus R_0_ values correlate with poorer hygiene and sanitation conditions. Thus, for treated CVID patients, we assume monthly death probabilities of up to 50 times (i.e., for the highest R_0_ value of 13) the monthly death probabilities calculated from the baseline survival curves (Table 3). We further factor in the possibility of IVIG-treatment lapses using the monthly income level-dependent lapse probabilities in Table 3, assuming these represent independent events and include both failure to receive IVIG during a month or reduced quality of the polio or other antibodies. If an IVIG-treatment lapse occurs, then we assume the PID patient becomes subject to the untreated monthly death probability for the time of the lapse. Given that effective treatment depends on delivery through a completely functional health system, we assume lower treatment fractions in lower income levels, with some projected increase over time (Fig. 2b). During simulation, if the proportion of PID patients receiving treatment (with or without lapse) equals less than the assumed treatment fraction for a given month, then we randomly add PID patients to the pool of treated patients until the proportion treated is no longer smaller than the assumed treatment fraction.

### PID onset, diagnosis, OPV infections, and VAPP

Further events for all surviving PID patients include onset of PID symptoms and diagnosis and events related to OPV infection, including the 1) monthly probability of primary and secondary OPV infection that depends on poliovirus vaccine use (i.e., OPV-only, IPV/OPV, or 0 if no OPV use), age, income level, diagnosis status, treatment status, and serotype, 2) monthly progression of OPV infection that depends on the potential for long-term poliovirus excretion determined at birth, and 3) monthly probability of developing VAPP while infected, with an income-level dependent probability of fatal VAPP. When a primary OPV infection occurs during simulation (i.e., a clinical PID patient receives OPV), we independently sample the serotype(s) based on the serotype probabilities in Table 3, which implies some possibility of 2 or 3 concurrent serotypes. For secondary OPV infections, we assume only one serotype based on the probabilities in Table 3. We assume probabilities of the serotype(s) excreted based on limited evidence from the iVDPVs isolated to date (Table 3) [18]. After OPV2 cessation, removal of the serotype 2 component implies no new creation of iVDPV2s (i.e., unless introduced for outbreak response or through an unintentional or intentional event). We assume no change in the overall rate of OPV infections after OPV2 cessation, with new infections involving serotypes 1 and/or 3 according to the same relative likelihood of serotypes 1 and 3 as before OPV2 cessation (Table 3). Similar to the very low estimated VAPP rate (i.e., approximately 1 per million) for first poliovirus infections in immunologically competent individuals [42], VAPP also represents a rare event for PID patients, with 4 CVID and 33 oPID patients reported with VAPP in the US during the 23-year period 1975–1997 [53]. Assuming approximately 400 annual PID births [42] in the US and that 20 % of PID patients represent CVIDs (Table 3), this translates into VAPP rates of 2200 and 4500 per million first infections in CVID and oPID patients, respectively. To translate these into monthly probabilities of VAPP given OPV infection, we assume that the GPEI identified all paralytic long-term poliovirus excretors that occurred during the last 5 years (i.e., 26 paralytic long-term excretors during 2009–2013) [18]. We then multiply the estimated rates above to obtain the same cumulative number of paralytic poliovirus excretors during 2009–2013 in the model as reported, which makes the rates depend on all other model assumptions. This approach results in monthly VAPP probabilities of 0.004 and 0.008 for CVID and oPID patients, respectively. Thus, while VAPP clearly represents a very high risk for long-term excretors, VAPP onset may not occur until many years of the OPV infection if at all [34, 53] and one known chronic excretor continues to excrete after approximately 30 years without development of VAPP to date [57].

### Effect of PAVDs

With respect to the use of PAVDs, we assume that at least 40 % of long-terms excretors may clear the virus within 5 days if they receive a PAVD regimen, based on the results of a small clinical trial of pocapavir in immunocompetent subjects (Table 3) [15]. Assuming that immunodeficient patients would respond as well as immunocompetent subjects, 40 % represents a lower bound given that some subjects apparently resistant to the drug potentially acquired resistant virus from other subjects in the trial after clearing their initial infections [15]. To provide bounds on the potential impact of PAVDs, we considered an upper bound of 90 % drug effectiveness, reflecting either higher effectiveness of pocapavir or the potential effectiveness of combining multiple compounds. We further considered three scenarios of PAVD use: 1) the *status quo*, which assumes no availability of the PAVD and provides a baseline for comparison, 2) passive PAVD use, which assumes that 50 % of iVDPV excretors identified by previously experiencing and surviving VAPP receive PAVDs for one month in January, 2020, and 3) active PAVD use, which represents a hypothetical upper bound and assumes 90 % of all iVDPV excretors receive PAVDs for one month in January, 2020. Both PAVD scenarios further administer PAVDs according to the same fractions to any long-term excretors that develop paralysis after January, 2020. If an excretor receives PAVDs, we randomly determine whether the infection clears based on the effectiveness assumption (i.e., 40 % or 90 %) and assume this occurs within a month of the start of PAVD treatment.

To perform analyses that demonstrate the global iVDPV prevalence behavior from the DES model with or without PAVDs, we run 1000 stochastic iteration of the DES model for the different types of populations in the global model with appropriate monthly death probabilities and treatment fraction [23], and then aggregate the results.

## Results

**Fig. 3:**
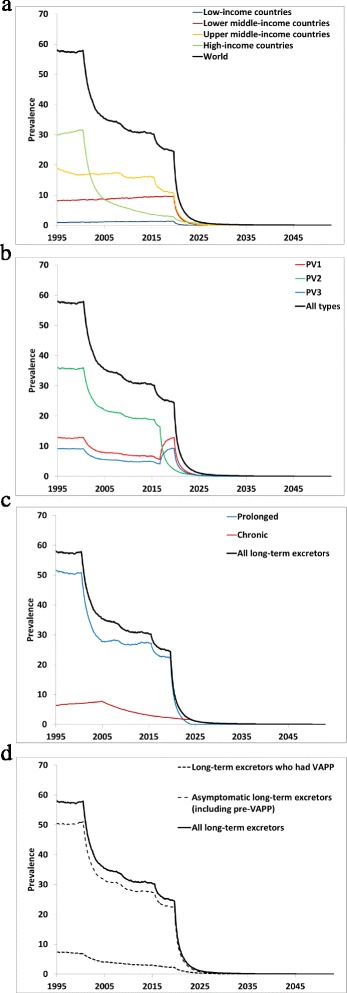
Prevalence of long-term iVDPV excretors in the absence of PAVD use, based on the monthly averages of 1,000 iterations of the DES model (**a**) Global and by income level (all serotypes, prolonged and chronic excretors, and clinical manifestations combined). **b** By serotype (all income levels, prolonged and chronic excretors, and clinical manifestations combined). **c** By prolonged vs. chronic excretors (all income levels, serotypes combined, and clinical manifestations combined). **d** By clinical manifestation (all income level, serotypes, and prolonged and chronic excretors combined)

Figure 3 shows the baseline prevalence (i.e., without PAVDs) of long-term iVDPV excretors by (a) income level, (b) serotype, (c) prolonged vs. chronic excretors, and (d) clinical manifestation. The estimated total global prevalence in 2013 equals approximately 30 long-term excretors, including approximately 27 active prolonged and 4 active chronic excretors. The number of prolonged excretors includes potential chronic excretors who did not yet progress past 5 years of excretion. In the context of high levels of continued OPV use to achieve eradication, the contributions of the relatively small number of long-term excretors to overall transmission of LPVs remains small and not easily observable. Thus, iVDPVs currently represent a relatively low and unnoticeable risk, except in countries that already switched to IPV-only routine immunization schedules for which any long-term excretors may represent a source of exposure to LPV. This includes high-income countries that maintain very high routine immunization coverage and benefit from relatively low R_0_ values such that any transmissions that may occur will die out.

As suggested in Fig. 3a, countries in the upper middle-income group, which includes about 2.5 billion people in 2013 (nearly 50 % of them in China), account for an estimated 16 total long-term excretors (52 % of the global estimate). Thus, while the high-income countries include more long-term excretors per capita during OPV use, they represent only approximately 1.2 billion people and the majority of these countries stopped all OPV use years ago, which already led to a decline in their long-term excretor prevalence.

As suggested in Fig. 3b, after OPV2 cessation, the prevalence of long-term serotype 2 excretors drops sharply, but serotype 1 and serotype 3 excretors increase because the first OPV infection now always occurs with one of these two serotypes. Before cessation of all OPV serotypes in 2019, prolonged excretors (including potential chronic excretors who excreted between 0.5-5 years) attain approximately seven times the prevalence of chronic excretors (Fig. 3c). However, after OPV cessation of all serotypes, not surprisingly the prevalence of prolonged excretors drops quickly, while a few chronic excretors continue to exist for over a decade. Most of the chronic iVDPV excretors after OPV cessation of all serotypes reside in upper middle- and high-income countries, with the prevalence in low- and lower middle-income countries dropping below the level of high-income countries within a few years of OPV cessation of all serotypes. Despite the relatively high monthly VAPP rates continuing throughout each long-term infection (Table 3), long-term excretors with VAPP still account for only approximately 10 % of all long-term excretors (Fig. 3d) because the remainder either will recover or die before VAPP or will not experience VAPP until later during their infection.

The results in Fig. 3 correspond to an average cumulative incidence of approximately 200 long-term iVDPV excretors during 2009–2013, compared to 33 reported by the GPEI during this period [18]. Given our approach for estimating VAPP rates, we obtain approximately the same number of paralytic long-term excretors as reported (i.e., 30 vs. 26), while the model estimates approximately 170 asymptomatic long-term excretors compared to 7 reported asymptomatic long-term excretors. Overall, the model suggests a global prevalence of approximately 30,000 PID patients in January, 2015 with suspected immunodeficiencies that may lead to longer times required to clear poliovirus infections. Combined with the 30 estimated current long-term excretors, this translates into a rate of approximately 0.001 long-term excretors per PID patient, consistent with the rate of approximately 0.001 observed among 978 PID patients screened for long-term poliovirus excretion shown in Table 1.

Given the importance of chronic excretors for long-term poliovirus risk management, we further compared the model results with the limited evidence about chronic excretors. The model estimates global CVID prevalence in 2015 of approximately 14,000 patients, compared to approximately 8,000 CVID patients known to a large network of PID treatment centers in 78 countries [40, 58]. This suggests a large number of unidentified CVID patients globally. However, significant uncertainty about the true global prevalence [38, 39] complicates verification of this model result. The data from chronic excretors identified since 1970 sum to approximately 53 person-years of chronic infection (i.e., not including the first 5 years of infection during the prolonged excretion time) and suspected chronic excretors detected through environmental surveillance of another 95 person-years (assuming a rate of 1.1 % VP1 divergence per year), for a total of almost 150 person-years of detected chronic infection. The 1,000 stochastic iterations of the DES model yield an average of approximately 200 person-years of chronic infection. The difference reflects the expected large proportion of asymptomatic chronic excretors (Fig. 3d) and the lack of systematic identification of chronic excretors.

**Fig. 4:**
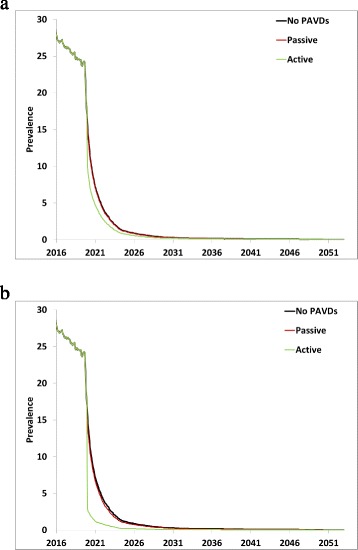
Impact of PAVD use on iVDPV prevalence for different PAVD use scenarios starting on January 1, 2020, based on the first 100 stochastic iterations of the DES model. **a** Lower bound on effectiveness of a single PAVD compound (i.e., assuming 40 % of recipients recover from infection). **b** Hypothetical upper bound on effectiveness of one or more PAVD compounds (i.e., assuming 90 % of recipients recover from infection)

Figure 4 shows the impact on iVDPV prevalence from the potential use of a PAVDs with low drug effectiveness of 40 % in clearing the infection (Fig. 4a) or high drug effectiveness of 90 % (Fig. 4b) for both PAVD use scenarios. Consistent with the low proportion of symptomatic iVDPV excretors in the model, relying on the occurrence of VAPP in PID patients to identify long-term excretors and treating 50 % of them leads to a negligible reduction in the prevalence, particularly if coupled with low drug effectiveness (Fig. 4a, red curve overlapping the black curve). The hypothetical scenario of active PAVD use with 90 % of all long-term excretors receiving PAVDs suggests potentially larger benefits, especially for high drug effectiveness (Fig. 4b, green curve). However, this scenario would require significant efforts to scale-up screening of PID patients in order to treat them. With the lower bound of PAVD effectiveness of 40 %, the active PAVD use scenario attains a 15 % reduction in iVDPV prevalence 10 years after OPV2 cessation. In contrast, with the upper bound of PAVD effectiveness of 90 %, the same level of PAVD use results in a 79 % reduction in iVDPV prevalence 10 years after OPV2 cessation.

## Discussion

As we enter the polio endgame, modeling can provide important insights about the different risks that may occur, which may help to identify opportunities to manage them. This reanalysis of iVDPV risks suggests that they may represent a larger concern after OPV cessation than previously recognized, although significant uncertainty remains. The estimated current prevalence of long-term excretors remains higher than the base case for our prior analysis based on the zero observed long-term excretors in screening studies available at the time, but lower than the prior upper bound estimate [2], reflecting the current non-zero observation of long-term excretors in more recent screening studies (Table 1) and our efforts to account for missing long-term excretors in those studies [52]. The proportion of PID patients who may develop prolonged or chronic excretion nevertheless remains a major uncertainty. Improved treatment of immunocompromised individuals continues to increase their survival, with treatment strategies increasingly transferred to relatively lower-income countries, which also contributed to the overall higher prevalence than previously estimated for these countries [2]. The insufficient data and uncertainty imply limitations of our model and results. For example, insufficient data exist to model the properties of each PID defect in the combined oPID category, but important differences certainly exist. More serious forms of PID (e.g., SCID) may imply a greater probability of long-term poliovirus excretion, but also lead to reduced survival, particularly in developing countries. However, treatment of SCID patients with bone marrow transplantation may also make these patients effectively immunocompetent and remove them from the risk pool altogether. In contrast, patients with milder PID defects with longer survival (e.g., hypogammaglobulinemia) probably recover spontaneously from poliovirus infections relatively early during prolonged replication. Combining all oPIDs without a correlation between survival and probability of long-term infection given OPV exposure represents the conservative approach and may overestimate prolonged excretion.

Other significant uncertainties also impact the risk estimates, which could lead to higher or lower estimates. The relatively high assumed probabilities of potential long-term excretion of 1 % (prolonged) or 0.5 % (chronic) (Table 3) used to adjust for missing excretors based on the limited cross-sectional screening studies may contribute to overestimation of the model because they exceed the observed rates of 0.1 % (prolonged) and 0 % (chronic). In contrast, our model may underestimate the current prevalence of long-term excretors in high-income countries by assuming that all high-income countries that use IPV-only as of 2013 stopped IPV use as early as the year 2000 (Table 2), while in reality some countries (e.g., Japan) continued to use OPV-only until recently. In addition, almost no data exits to quantify PID survival in developing countries and therefore our assumed survival rates could over- or underestimate the risk. Although we present the average results of 1000 realizations of the model, the stochastic events modeled could lead to very different potential futures and even with all of the new information collected since our prior analysis [2], iVDPV risks remain highly uncertain and of significant concern for polio endgame risk management. Further studies should provide additional information that may help to reduce uncertainties.

Table 4 highlights high-impact and reasonably feasible research opportunities to reduce uncertainties in future iVDPV risk estimates that we identified through the model development process. The opportunities include research to reduce uncertainty about key drivers of the prevalence of iVDPVs after OPV cessation, such as retrospective analysis of global PID registries to better characterize PID survival in low- and middle-income countries, expanded (and if feasible) longitudinal poliovirus screening of PID patients to better estimate the probability of long-term excretion for PID patients, and clinical trials with PAVDs involving long-term excretors to determine the PAVD effectiveness in clearing poliovirus infection. Other research opportunities may address specific questions identified through the model development process or provide better estimates of the bounds of current true global long-term poliovirus excretor prevalence. With the long lead time for research, development, and implementation of risk management strategies and the long observation period required for longitudinal screening, the research opportunities we identified represent urgent, although in some cases potentially costly priorities. However, the potential consequences of an iVDPV-associated outbreak in a developing country after OPV cessation in the absence of a tested outbreak response strategy beyond the first few years after OPV cessation will likely justify further investments to improve iVDPV risk management. Some lower-cost research opportunities, such as more systematic reporting of timelines of events associated with long-term excretors and continued follow-up [59] beyond recovery from infection, may help resolve a number of specific questions identified through our modeling process. In addition to the questions in Table 4, we identified many other specific theoretical questions and practical issues not addressable by feasible, short-term research. For example, a better understanding of the immunological mechanisms of long-term poliovirus excretion and recovery would help inform model inputs and address the question of reinfection. Assessing the impact of iVDPV prevalence on risks after OPV cessation requires integration of the results from the iVDPV prevalence model with a global poliovirus transmission model [23]. In this context, the transmissibility of iVDPVs compared to cVDPVs or WPVs represents an important uncertainty that will significantly affect the risk of any transmission from long-term excretors over time. While we cannot directly estimate R_0_ values for poliovirus, environmental sampling and expanded stool surveys around known excretors may provide data to inform assumptions going forward.

**Table 4:** High-impact research opportunities identified to address key questions and reduce uncertainty in future iVDPV risk estimates

Research opportunities	Research questions
Retrospective analysis of global PID registries	- What are the survival prospects of individuals in different PID categories in different income levels (particularly CVIDs) and how are they changing over time?
	- How does IVIG treatment affect PID survival?
Expanded and longitudinal screening of PID patients for poliovirus (with reporting of IVIG treatment status for screened PID patients)	- What fraction of patients with different PIDs (CVID, SCID, others) will develop prolonged and chronic excretion if infected with OPV?
	- How many prolonged and chronic excretors currently exist globally?
	- How does IVIG treatment affect the probability of developing a long-term infection if infected with OPV?
Clinical trials with PAVDs involving long-term excretors	- How effective are individual and combined PAVDs in clearing poliovirus infections?
Systematic detailed reporting of timelines of events for all known long-term excretors	- Does a PID patient who spontaneously recovered from a long-term infection develop sufficient immunity to prevent future long-term infections of the same or other serotypes?
	- How does IVIG treatment affect the probability of developing a long-term infection if infected with OPV?
	- What is the current survival and excretion status and the estimated time of infection, recovery (if applicable) and death (if applicable) for each known long-term excretor?
Continued follow-up of all identified long term excretors (including after spontaneous recovery from infection )	- Does a PID patient who spontaneously recovers from a long-term infection develop sufficient immunity to prevent future long-term infections of the same or other serotypes?
	- Do frequent concurrent enteric infections result in an increased probability of spontaneous recovery from long-term poliovirus infections?
Intensification of searches for PID patients causing apparent iVDPVs isolated from the environment	- Who are the sources of apparent iVDPVs isolated from the environment?
	- What are the bounds on current chronic excretor prevalence in countries that stopped using OPV?
Expanded environmental surveillance for apparent iVDPVs	- What are the bounds on current chronic excretor prevalence?
	- Can iVDPVs transmit widely?
Modeling of iVDPV introductions into the general population after OPV cessation	- What are the expected consequences of prolonged and chronic excretion beyond OPV cessation?
Expanded stool sampling around known long-term excretors	- Are iVDPVs as transmissible as cVDPVs and WPVs?

After OPV cessation, efforts to identify chronic excretors will represent a necessity for risk management. If effective PAVDs exist, then treating chronic excretors both to protect them from potentially developing VAPP and to protect the rest of the population from exposure to LPVs will offer an important strategy to reduce risk. Efforts to identify patients in the presence of an effective treatment strategy will most likely benefit from the potential to prevent future VAPP in these patients and may support widespread testing of PIDs for poliovirus excretion, including asymptomatic patients. Development of a second compound (or more) will potentially help to both motivate a scale-up of screening for long-term poliovirus excretors and achieve more impact of widespread PAVD use on the risk of iVDPVs after OPV cessation. In contrast, in the absence of any PAVDs, risk management efforts may focus on patient education aimed at asking the patients to limit their exposure to others (i.e., self-isolation to some degree). This will represent a challenge given the asymptomatic nature of most infections and the lack of incentives for patient participation.

OPV cessation will ultimately stop the creation of new long-term excretors, but it will take time for the chronic excretors that exist at the time of OPV cessation to stop excreting. Countries and the GPEI will need to recognize that in a world without high levels of population immunity to transmission derived from use of LPVs, iVDPVs will represent an important potential source for re-introduction of poliovirus that requires active risk management.

## Conclusions

Further research into PID incidence, survival, and long-term excretion would reduce important uncertainties associated with the risk from long-term poliovirus excretors. Efforts to develop a second polio antiviral compound to increase PAVD effectiveness and/or to maximize the identification and treatment of affected individuals represent important risk management opportunities for the polio endgame.

## Abbreviations

cVDPV: Circulating vaccine-derived poliovirus
CVID: Common-variable immunodeficiency disease
DES: Discrete-event simulation
GPEI: Global polio eradication initiative
IPV: Inactivated poliovirus vaccine
iVDPV(1,2,3): Immunodeficiency-associated vaccine-derived poliovirus (serotype 1, 2, or 3, respectively)
IVIG: intravenous immunoglobulin
LPV: Live poliovirus
oPID: Other PID
OPV: Oral poliovirus vaccine
OPV2 cessation: Globally-coordinated cessation of all serotype 2-containing OPV
PAVD: Polio antiviral drug
PID: Primary immunodeficiency disease
PV(1,2,3): Poliovirus (serotype 1, 2, or 3, respectively)
R_0_: Basic reproduction number
SCID: Severe combined immunodeficiency disease
SIA: Supplemental immunization activity
VAPP: Vaccine-associated paralytic poliomyelitis
WPV(1,2,3): Wild poliovirus (serotype 1, 2, or 3, respectively)
